# H2A.Z marks antisense promoters and has positive effects on antisense transcript levels in budding yeast

**DOI:** 10.1186/s12864-015-1247-4

**Published:** 2015-02-19

**Authors:** Muxin Gu, Yanin Naiyachit, Thomas J Wood, Catherine B Millar

**Affiliations:** Faculty of Life Sciences, University of Manchester, M13 9PT Manchester, UK

**Keywords:** Antisense transcript, Chromatin, H2A.Z, Histone variant, Htz1

## Abstract

**Background:**

The histone variant H2A.Z, which has been reported to have both activating and repressive effects on gene expression, is known to occupy nucleosomes at the 5' ends of protein-coding genes.

**Results:**

We now find that H2A.Z is also significantly enriched in gene coding regions and at the 3' ends of genes in budding yeast, where it co-localises with histone marks associated with active promoters. By comparing H2A.Z binding to global gene expression in budding yeast strains engineered so that normally unstable transcripts are abundant, we show that H2A.Z is required for normal levels of antisense transcripts as well as sense ones. High levels of H2A.Z at antisense promoters are associated with decreased antisense transcript levels when H2A.Z is deleted, indicating that H2A.Z has an activating effect on antisense transcripts. Decreases in antisense transcripts affected by H2A.Z are accompanied by increased levels of paired sense transcripts.

**Conclusions:**

The effect of H2A.Z on protein coding gene expression is a reflection of its importance for normal levels of both sense and antisense transcripts.

**Electronic supplementary material:**

The online version of this article (doi:10.1186/s12864-015-1247-4) contains supplementary material, which is available to authorized users.

## Background

Chromatin components are key regulators of global gene expression. H2A.Z is a highly conserved histone variant that replaces H2A in a subset of nucleosomes, most prominently those that flank the transcription start sites (TSSs) of protein-coding genes (reviewed in [1]). TSS-adjacent H2A.Z localization is suggestive of a role in transcriptional regulation and indeed H2A.Z has been implicated in gene regulation in multiple organisms [2-5]. As H2A.Z is essential for normal development [6-8] and its over-expression is associated with poor patient prognosis in human cancers [9,10], it is important to understand how H2A.Z contributes to the regulation of gene expression.

Previous studies linking H2A.Z to transcription have primarily focused on protein-coding genes. It has recently become clear that eukaryotic transcriptomes are a complex mixture of coding and non-coding transcripts, with many transcripts being rapidly turned over by RNA-processing machinery such as the exosome (reviewed in [11,12]). In *S. cerevisiae*, such cryptic unstable transcripts often originate from the 5′ nucleosome-depleted-region (NDR) of a downstream tandemly arranged gene and can be detected in the absence of the exosome catalytic component Rrp6 [13,14]. In this study, we used yeast strains lacking Rrp6 to compare H2A.Z occupancy to global transcription and found that H2A.Z occupies the 3′ ends of protein-coding genes in addition to its well-known enrichment at their 5′ ends. H2A.Z is co-localised with other active histone modifications at the 3′ end of genes, at sites that match the start sites of non-coding transcripts transcribed in the antisense orientation relative to the sense protein-coding genes. The deletion of H2A.Z results in the down-regulation of antisense transcripts that normally have H2A.Z in their promoters. This novel association between H2A.Z and antisense transcripts differs fundamentally from the previously described role of H2A.Z in supressing antisense transcripts in fission yeast [15]. Our findings indicate that H2A.Z is a general marker of TSSs and suggest that some apparently indirect effects of H2A.Z deletion on the expression of protein-coding genes whose promoters do not contain H2A.Z are mediated through effects on an antisense transcript.

## Results and discussion

### H2A.Z is significantly enriched at the 3′ ends of genes

**Figure 1:**
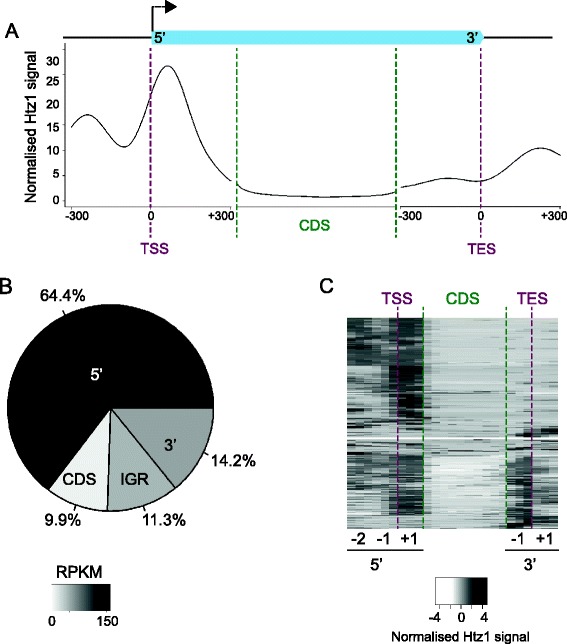
**About one-third of Htz1 is localised outside of TSSs. A**. Average profile of Htz1 at protein-coding genes, with an average transcript depicted by a light blue box. Genes were aligned according to their TSSs or transcription end sites (TESs) and the average levels of Htz1 (reads per million sequenced reads per gene) were calculated for each base pair. For relative alignments of coding regions (CDS; region >300 bp down/up-stream of TSS/TES), all transcripts were stretched or compressed to a constant length and then the average Htz1 level was found at each relative position. **B**. The percentages of Htz1 signal located at the 5' end (−300 to +300 relative to TSS), CDS, and 3' end (−300 to +300 relative to TES) of protein-coding genes are shown, along with enrichment in other intergenic regions (IGR; > 300 bp away from a protein coding gene). The darkness of the colour for each category reflects the length-normalised density of Htz1 signal in reads per kilobase per million mapped reads (RPKM). **C**. Hierarchical clustering of Htz1 binding profiles, highlighting the clusters of genes with Htz1 enrichment at TSSs, CDSs and TESs. A 25-dimensional vector was generated for each transcript, comprising nine 50-bp windows corresponding to the −2, −1 and +1 nucleosomes around the TSS (5' -2, 5' -1 & 5' +1), 10 windows for the coding sequence (CDS) and six 50-bp windows for the nucleosomes flanking the TES (3' -1; 3' +1). Clustering was done using Euclidean distances between the vectors. Data in this figure were generated from one biological replicate, but are essentially identical to a second wild-type biological replicate (Additional file 5: Table S1).

Numerous ChIP-chip and ChIP-seq studies, in various organisms and cell types, have revealed high enrichment of H2A.Z at the promoters of protein-coding genes. In this study we used highly specific antibodies ([16]; Additional file 1: Figure S1A,B) to endogenous untagged budding yeast H2A.Z (Htz1) in ChIP-seq experiments to assess whether there were other, less obvious, patterns of Htz1 enrichment that had hitherto gone un-noticed. We found that besides the high enrichment of Htz1 at the 5′ ends of protein coding genes, Htz1 is also enriched at the 3′ ends and inside coding sequences (CDSs; Figure 1A, B). ChIP-seq analysis in an *htz1∆* strain confirmed that these signals are all specific to Htz1 (Additional file 1: Figure S1C, D). Quantification of ChIP-seq signals revealed that about one-third of the Htz1 signal is found outside of the 5′ ends of protein-coding genes, at 3′ ends, intergenic and intragenic regions (Figure 1B). Notably, the 3′ ends of genes harbour nearly 15% of the Htz1 signal and the density of Htz1 occupancy (*i.e.* length-normalised occupancy) is higher at 3′ ends than at intra- and inter-genic regions (Figure 1B). Out of 5143 protein coding genes, 1025 have a high level of Htz1 at their 3′ end, while 2506 have high Htz1 levels at their 5′ ends. Clustering of the Htz1 distributions along genes revealed that some have Htz1 only at their 5′ or 3′ ends while others have Htz1 at both 5′ and 3′ ends (Figure 1C). Although prior studies have primarily focused on the TSS-adjacent occupancy of H2A.Z, Coleman-Derr *et al.* have observed enrichment of H2A.Z in gene bodies and 3′ ends in *Arabidopsis thaliana* [17], in agreement with our findings in *S. cerevisiae*.

### Peaks of H2A.Z at the 3′ ends of genes correlate with other active histone marks

**Figure 2:**
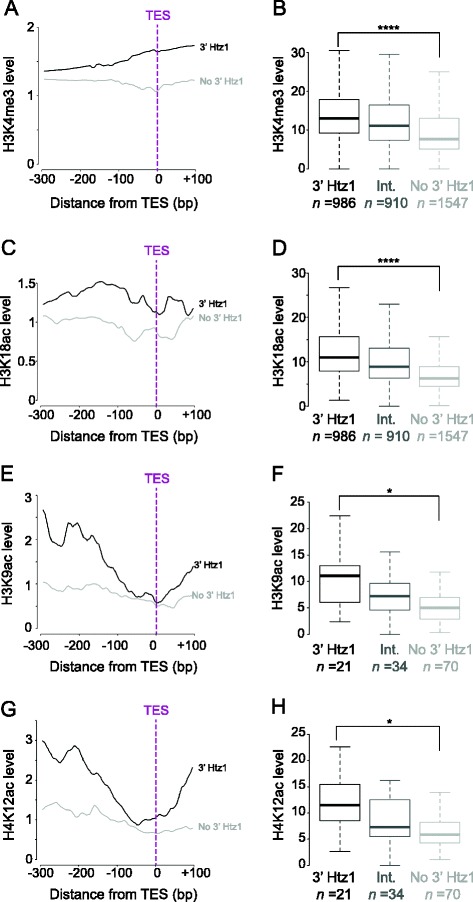
**Htz1 at 3′ ends of genes co-localises with active histone modifications.** Histone modifications known to occupy active promoters include H3K4me3 [19], H3K18ac [20]*,* H3K9ac and H4K12ac [18]. H4K4me3 and H3K18ac data are from the whole genome; H3K9ac and H4K12ac are restricted to chromosome 3. **A**, **C**, **E**, **G**. Profiles of histone modifications at the 3' ends of genes with 3' Htz1 enrichment (black lines) or without Htz1 enrichment (grey lines). All four active histone marks are enriched upstream of the TESs of genes that have high 3' Htz1 occupancy. **B**, **D**, **F**, **H**. Boxplots of the distributions of histone modification levels in genes with high 3' Htz1 enrichment (3' Htz1; black boxes), without Htz1 enrichment (no 3' Htz1; light grey boxes) or with intermediate levels of 3' Htz1 (Int.; medium grey boxes). The number of genes in each category is indicated. H3K4me3 (*p* = 4.0 x 10^−29^), H3K18ac (*p* = 8.0 x 10^−57^), H3K9ac (*p* = 6.4 x 10^−3^) and H4K12ac (*p* = 1.2 x 10^−2^) are significantly higher on genes with high 3' Htz1. *p ≤ 0.05, ****p ≤ 0.0001. The *p-*values were obtained using two-tailed *t-*tests.

As antisense transcription is widespread in budding yeast [13,14], we hypothesised that the prominent peaks of Htz1 at the 3′ ends of genes could reflect the TSSs of antisense transcripts initiating in these regions. If this were the case, the 3′ peaks of Htz1 would be expected to co-localise with histone modifications that are normally present at actively transcribed promoters. We examined levels of histone modifications that are known to be enriched in the promoter regions of active genes [18] and observed that the 3′ regions with high Htz1 occupancy are significantly enriched for active histone marks, including H3K4me3, H3K18ac, H3K9ac and H4K12ac (Figure 2). The correlations we observe between 3′ Htz1 occupancy and other marks of active promoters are consistent with these 3′ peaks of Htz1 potentially being promoters of antisense transcripts.

### H2A.Z at the 3′ end of genes marks the start of antisense transcripts

**Figure 3:**
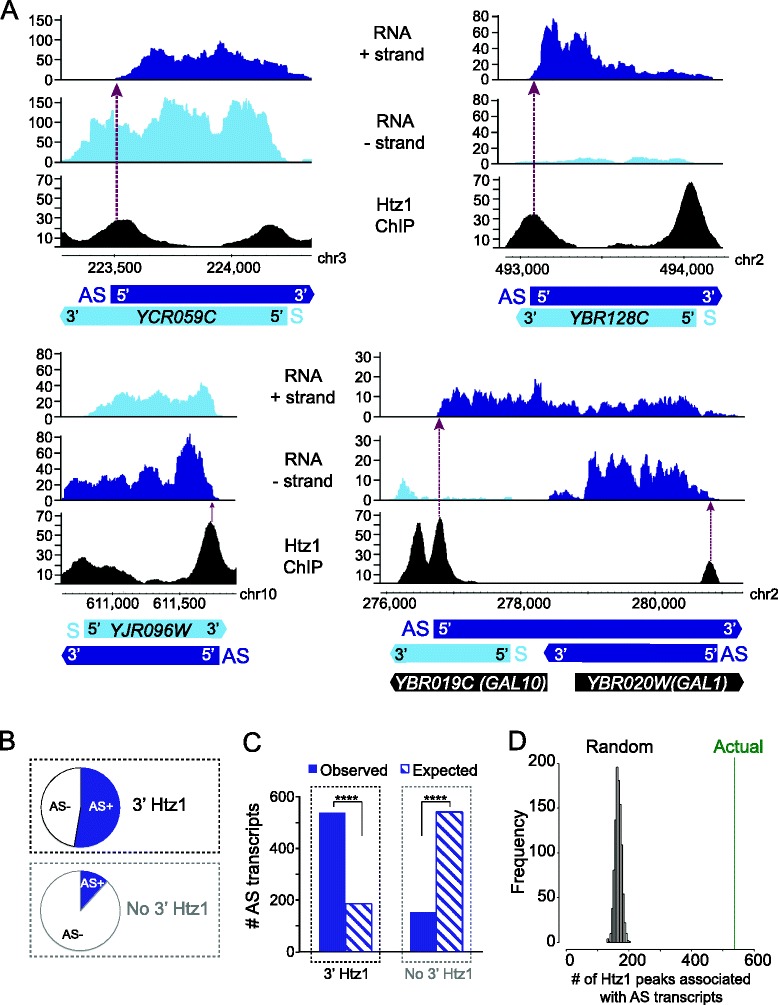
**Htz1 at 3′ ends marks the start of antisense transcripts. A**. Transcripts derived from the + and – strands are shown, along with Htz1 occupancy over the same regions for the protein coding genes *YCR059C, YBR128C, YJR096W, YBR019C and YBR020W*. Antisense (AS) transcripts are coloured dark blue and sense (S) transcripts light blue. Htz1 ChIP peaks at the 3' ends of protein-coding transcripts are co-incident with the 5' ends of antisense transcripts, as indicated by the arrows. **B**. Fraction of genes with (top) or without (bottom) 3' Htz1 that have associated antisense transcripts. 537 (52%) out of 1025 genes that have a high level of 3' Htz1 are associated with antisense transcripts whereas only 155 (5%) out of 3010 genes with no 3' Htz1 have antisense transcripts. **C**. Number of 3' end regions associated with antisense transcripts. Out of the 1025 genes whose 3' ends are occupied by high levels of Htz1, 537 are associated with antisense transcripts, which is significantly higher than the expected 185 (****p ≤ 0.0001 (1.7 x 10^−187^), Fisher’s exact test), whereas out of the 3010 genes whose 3' ends are depleted of Htz1 only 155 are associated with antisense transcripts, which is significantly lower than the expected 542 (****p ≤ 0.0001 (3.6 x 10^−184^), Fisher’s exact test). **D**. Comparison of the number of 3' Htz1 peaks associated with antisense transcripts (green line) to the distribution of random 3' regions (black bars) that co-localise with antisense transcripts. 987 regions of 150 bp were drawn randomly from the 3' ends of genes and the number co-localising with antisense transcripts was calculated. This randomisation was performed 1000 times to produce the histogram showing the distribution of random peaks that co-localise with antisense transcripts. The actual association of Htz1 with antisense transcripts is highly significant (*p* = 0).

To directly test whether 3′ peaks of Htz1 correspond to the start sites of antisense transcripts, we next compared Htz1 occupancy to strand-specific transcript data generated from a strain lacking the exosomal RNA-degrading enzyme, Rrp6. We focused on the Htz1 signal located upstream rather than downstream of the transcription end site (TES) in order to avoid Htz1 signal that is potentially derived from the 5′ promoter of the adjacent gene in the compact *S. cerevisiae* genome. We observed a total number of 1076 antisense transcripts in the *rrp6Δ* strain and the Htz1 peaks at the 3′ ends of genes frequently correspond to the 5′ ends of antisense transcripts (see Figure 3A for examples). Over 50% of genes with Htz1 occupancy in their 3′ end have a detectable antisense transcript in the *rrp6Δ* strain, while only 5% of genes without 3′ Htz1 have an antisense transcript (Figure 3B). The frequency of antisense transcripts in 3′ regions that are enriched for Htz1 is significantly higher than expected *(p* = 1.7 × 10^−187^) and, conversely, the frequency is significantly lower than expected (*p* = 3.6 × 10^−184^) in 3′ regions lacking Htz1 (Figure 3C). Randomisation of the Htz1 peaks at the 3′ ends of all genes generated a distribution of peaks coinciding with antisense transcripts that is markedly lower than the actual number we observe, with a false discovery rate close to zero (Figure 3D). We have focused on peaks at the 3′ end of genes because of this striking overlap but we also observed that a lower percentage of coding region Htz1 peaks overlap with the start sites of antisense transcripts (Additional file 2: Figure S2B). Our findings implicate Htz1 as a general marker of TSSs, for both sense and antisense transcripts. As at the 5′ ends of protein-coding genes, where Htz1 is widespread but not at every gene promoter (Figure 3A; [16,21-24]), Htz1 is not a ubiquitous feature of the 5′ ends of antisense transcripts. This is comparable to other chromatin components that have been implicated in regulating antisense transcript levels, including the ATP-dependent remodeler Isw2 [25], the histone deacetylase complexes Rpd3S [26] and Set3C [27], and methylation of H3K4 [28], which also mark or regulate a sub-set of antisense transcripts.

### H2A.Z is important for antisense transcript levels

**Figure 4:**
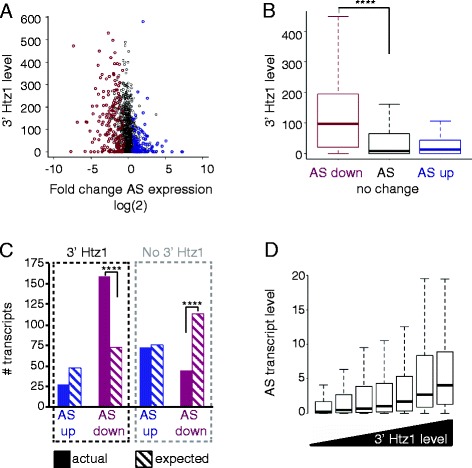
**Htz1 affects antisense transcript levels. A**. Comparison of differential antisense (AS) transcript levels in *rrp6∆htz1∆∆* versus *rrp6∆* to Htz1 levels at the 3' ends of genes. Each transcript is shown as an open circle, with its 3' Htz1 level measured by ChIP-seq being the y-value and its fold change of expression in the *rrp6∆htz1∆* strain shown as its x-value. Significantly up- and down-regulated transcripts are coloured in blue and magenta respectively. **B**. Boxplots of the distributions of 3' Htz1 levels for down- (*n* = 255) and up- (*n* = 169) regulated antisense transcripts show that down-regulated antisense transcripts are significantly enriched for Htz1 (****p ≤ 0.0001 (2.9 x 10^−21^); two-tailed *t-*test) compared to transcripts whose expression doesn’t change (*n* = 3019). **C**. Actual (solid bars) and expected (hatched bars) number of up-/down-regulated antisense transcripts with and without 3' Htz1. Down-regulated antisense transcripts with 3' Htz1 are significantly more numerous than expected (****p ≤ 0.0001 (1.0 x 10^−31^); Fisher’s exact test), whereas those without 3' Htz1 are significantly fewer than expected (****p ≤ 0.0001 (1.6 x 10^−21^); Fisher’s exact test). **D**. Enrichment of Htz1 at the 3' end of genes positively correlates with the level of the associated antisense transcript. Genes were classified into bins of seven quantiles according to 3' Htz1 level. The distribution of antisense transcript levels are plotted for each bin, arranged from low 3' Htz1 (left) to high 3' Htz1 (right).

To test whether Htz1 binding at the 3′ ends of genes contributes to regulation of antisense transcripts, we examined the antisense transcriptomes in *rrp6∆* and *rrp6∆htz1∆* cells. To establish which transcripts are likely to be regulated directly by Htz1, we compared changes in transcript levels in the absence of Htz1 to Htz1 occupancy (Figure 4A). This analysis revealed that the majority of affected antisense transcripts are enriched for Htz1, consistent with a direct effect for Htz1 in regulating these transcripts although indirect effects are also formally possible. Notably, down-regulated antisense transcripts have significantly higher Htz1 occupancy at their 5′ ends (*i.e.* the 3′ ends of genes) while promoters of up-regulated antisense transcripts are less enriched for Htz1 (Figure 4B). Quantification of up- and down-regulated antisense transcripts revealed that the down-regulation of antisense transcripts occurs significantly more often than expected for genes with 3′ Htz1 occupancy (*p* = 1.0 × 10^−31^; Figure 4C). In addition, the occupancy of Htz1 at the 3′ ends of genes is positively correlated with antisense transcript levels in the wild-type strain (Figure 4D). Together, these data are consistent with a predominantly activating effect for Htz1 at the promoters of antisense transcripts. While we also observe a predominantly activating effect for Htz1 at the 5′ ends of genes on sense transcripts, the effect is weaker than for antisense transcripts, as the *p-*values are markedly higher than at the 3′ ends (Additional file 3: Figure S3A, B, C).

### Antisense transcripts that depend on H2A.Z are primarily transcribed from tandemly arranged genes

**Figure 5:**
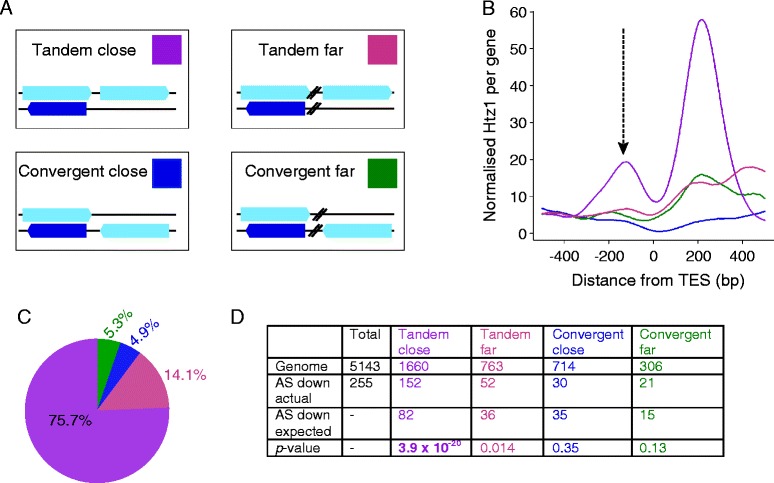
**Htz1 predominantly affects tandemly arranged sense/antisense genes. A**. Genomic arrangement of the 4 gene categories, with sense transcripts depicted as light blue boxes on the + or – strand and antisense transcripts shown as dark blue boxes. Tandem or convergent refers to the sense transcripts. Close genes have <300 bp between them, while the distance is >300 bp for the “far” genes. The coloured box for each category is the key for **B**, **C** & **D**. **B**. Htz1 levels at the 3' ends of genes, aligned by TESs and coloured according to A. The arrow indicates the 3' peak upstream of the TES that we have focused on. Convergent genes have less Htz1 associated with their 3' ends, with “tandem close” genes having the highest Htz1 levels. **C**. Quantification of the 3' Htz1 signal shows that the majority of 3' Htz1 is found at tandem genes. **D**. Htz1 affects a significant number of tandem close antisense transcripts. The number of antisense transcripts down-regulated in *rrp6∆htz1∆* was compared to the number in that category in the genome for each of the 4 gene arrangements. The *p* values are derived from Fisher’s exact tests.

H2A.Z has previously been implicated in the regulation of antisense transcript levels in the fission yeast *S. pombe*, although in that case H2A.Z supresses antisense transcript levels specifically at convergently transcribed genes by preventing transcriptional read-through [15]. As H2A.Z was not observed at the 3′ ends of genes in that study and had the opposite effect on antisense transcript levels, this previously described role of H2A.Z appears mechanistically different from what we have observed in budding yeast. Nonetheless, we checked whether there was a similar tendency of Htz1 to regulate antisense transcripts at convergent genes in budding yeast. We divided all intergenic regions into 4 classes depending on whether their flanking protein-coding genes were tandemly or convergently arranged and on the distance between the protein coding genes (Figure 5A). “Close” genes are separated by <300 bp, while “far” genes have >300 bp between their TSSs/transcription end sites (TESs). We found that most of the 3′ Htz1 signal is found in tandemly arranged genes rather than convergently arranged genes, and is particularly enriched in the “tandem close” category (Figure 5B, C; Additional file 4: Figure S4). To further investigate the relationship between gene organisation and the effect of Htz1 on antisense levels, we examined the number of genes in each category whose antisense transcripts are down-regulated in the absence of Htz1 (Figure 5D). This analysis also demonstrates a significant enrichment in the tandem close category (*p* = 3.9 × 10^−20^), indicating that Htz1 primarily affects antisense transcripts that are initiated from the promoters of tandemly arranged genes. The role of H2A.Z at the TSS of antisense transcripts in *S. cerevisiae* is therefore distinct from its previously described function in suppressing read-through antisense transcripts at convergent genes in *S. pombe*. This may reflect a difference in transcriptome organisation between *S. cerevisiae* and *S. pombe*, as the majority of antisense transcripts in *S. pombe* are generated by transcriptional read-through at convergent genes [29] while most antisense transcripts are derived from tandem genes in *S. cerevisiae* [13,14].

### Regulation of antisense transcripts by H2A.Z can affect sense transcript levels

**Figure 6:**
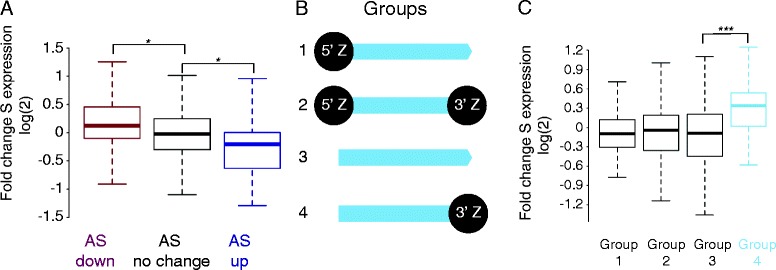
**The effect of Htz1 on antisense transcript levels impacts sense transcripts. A**. Genes with 3' Htz1 enrichment and an antisense transcript were divided into three groups based on whether their antisense transcript levels were unchanged (*n* = 794), up- (*n* = 28) or down- (*n* = 159) regulated in *rrp6∆htz1∆.* The boxplots show changes in sense (S) transcript levels for these groups. Down-regulated antisense transcripts have a significantly higher fold change in sense expression (*p ≤ 0.05 (0.02), two-tailed *t*-test) and conversely sense transcript levels are significantly decreased when antisense transcripts are up-regulated (*p ≤ 0.05 (0.05), two-tailed *t*-test). **B**. Classification of genes with both sense and antisense transcripts according to 5' and 3' enrichment of Htz1 was performed using thresholds for 5' and 3' enrichment based on average Htz1 occupancies at the 5' and 3' ends. Class 1 (*n* = 110) have only 5' enrichment; class 2 (*n* = 237) have 5' and 3' enrichment; class 3 (*n* = 75) are not enriched for Htz1 at either 5' or 3' ends; and class 4 (*n* = 34) have Htz1 only at the 3' end. **C**. Distributions of sense transcript fold changes in the *rrp6∆htz1∆* strain for each of the classes of genes illustrated in B. Sense transcripts are significantly up-regulated in group 4 genes that have Htz1 at their 3' ends only (***p ≤ 0.001 (7.5 x 10^−4^), two-tailed *t*-test).

Htz1 has previously been implicated in regulating sense transcripts and we now show that it affects antisense transcripts in budding yeast. Levels of sense and antisense transcripts at individual genes are generally anti-correlated (reviewed in [30,31]), which raises the question of whether the regulation of antisense transcripts by Htz1 affects sense transcript levels. We took genes with antisense transcripts and 3′ Htz1 occupancy, and examined the changes in sense transcript levels for genes where the antisense transcript either didn’t change, went up, or went down. Genes whose antisense transcript decreases in the absence of Htz1 show a corresponding increase in sense transcript levels (*p* = 0.02, Figure 6A). Conversely, the expression levels of sense transcripts corresponding to up-regulated antisense transcripts were decreased (*p* = 0.05, Figure 6A).

It is challenging to dissect the dependencies of sense/antisense transcript levels due to their inter-connected nature but the fact that Htz1 is more enriched at down-regulated sense and antisense transcripts leads us to propose a model in which the effects of Htz1 on down-regulated transcripts are direct and that up-regulated transcript levels generally result from an effect on the corresponding sense/antisense. To test this, we divided genes into 4 groups based on their Htz1-enrichment patterns (Figure 6B). Group 1 genes have Htz1 at their 5′ ends only and, based on our observations of sense transcript levels (Additional file 3: Figure S3), we would predict that levels of group 1 transcripts should decrease slightly when *HTZ1* is deleted. Although the median log (2) expression is lower than 0 in this group, it is not significantly lowered. The behaviour of Group 2 genes is unpredictable as they have Htz1 at both 5′ and 3′ ends, and changes at Group 3 genes (having no Htz1 at either 5′ or 3′ end) can be attributed to indirect effects. However, Group 4 genes (having Htz1 only at 3′ ends) are predicted to have up-regulated sense transcripts and indeed we find that Group 4 genes are significantly up-regulated (*p* = 7.5 x 10^−4^; Figure 5C). These findings are consistent with Htz1 having an effect on antisense transcripts that consequently affects sense transcript levels and may indicate that Htz1 is more important for the activation of antisense transcripts than sense transcripts.

## Conclusions

We find in this study that a significant proportion of Htz1 is located at the 5′ ends of antisense transcripts, and that Htz1 is required for normal levels of these non-coding transcripts in addition to its known role in regulating protein-coding genes. Comparison of strand-specific RNA-seq and ChIP-seq data shows that Htz1 occupancy has a predominantly activating effect on the promoters of antisense and sense transcripts. Previous work has described both activating and repressing roles for H2A.Z at individual genes in both yeast and mammalian cells (reviewed in [32]). While some genes that are up-regulated in *htz1Δ* have Htz1 at their promoters, these are relatively rare and most promoters of up-regulated transcripts have low Htz1 occupancy, arguing against a direct repressive effect of Htz1 on most transcripts, as previously observed by Li *et al.* [24] for protein-coding genes. However, up-regulation of protein coding genes that lack Htz1 in their promoters is not due to completely indirect effects, at least in some cases, but is mediated by Htz1 affecting levels of antisense transcripts generated from the 3′ ends of these genes. Mechanistically, some changes of sense transcript levels in *htz1Δ* strains have previously been ascribed to the aberrant activity of the SWR-C when Htz1 is absent [33]. We could not test whether Swr1 is responsible for the changes in AS levels in *rrp6Δhtz1Δ* because we were unable to generate strains triply mutated for *rrp6Δhtz1Δ* and *swr1Δ.*

The importance of H2A.Z for antisense transcription may explain some of its apparently conflicting effects on the expression of protein coding genes and highlights the need to study all transcripts derived from a locus in order to fully understand how genes are regulated.

## Methods

### Yeast genetics and molecular biology

Yeast strains were created using standard methods and are described in Additional file 5: Table S2. Cells for RNA extraction and ChIP were in harvested from log-phase cultures growing in SD medium. Total RNA was purified using the Ribopure Purification Kit (Life Technologies) and contaminating genomic DNA was removed by DNaseI digestion. Htz1 ChIP was performed essentially as described previously [34], using affinity purified custom αHtz1 (α660) antibodies [16] in a protocol optimised for maximal Htz1 recovery and extensive chromatin fragmentation by sonication. RNA and purified ChIP DNA were amplified for sequencing using the Illumina TruSeq stranded mRNA sample preparation kit or the Illumina TruSeq ChIP Sample Prep Kit. Libraries were sequenced on either an Illumina GAIIX or HiSeq 2000. ChIP-seq samples had an average of 12 million uniquely mapping reads, and RNA-seq samples had an average of 35 million reads (Additional file 5: Table S3). Data were generated from two independent biological replicates for each strain in each analysis (ChIP-seq/RNA-seq) and correlations between biological replicates were high in all cases (Additional file 5: Table S1 A, B).

### Sequence mapping

Sequence reads were mapped to the *S. cerevisiae* genome assembly sacCer1 using Bowtie version 0.12.9 [35], allowing up to 2 mismatches and no ambiguously mapped reads. Genomic coordinates for protein-coding transcripts were obtained from Xu *et al.* [14].

### ChIP-seq data processing

Background signal was set to 1.2 standard deviations above the mean of the ChIP:input ratios and then subtracted from the ChIP-seq signal. Where background was higher than the ChIP-seq signal, the value was set to zero. The final ChIP-signal at each base pair was normalized to the number of reads per million mapped reads.

### Strand-specific RNA-seq data processing

RNA-seq mapped reads were segregated into + and – strands. Normalisation was performed such that the total amount of sense-strand RNA was adjusted to 10^8^ arbitrary units and antisense RNA-seq levels were adjusted by the same factor. Transcript levels were then normalized per kilobase of transcript length.

### Data analysis

Regions that are 150 bp downstream of the TSS and upstream of the TES were used to quantify the 5′ and 3′-end enrichment of Htz1 respectively. Htz1 peaks were associated with a transcript if they had downstream RNA signals >3-fold higher than upstream signals. The nature of the associated transcripts (i.e. sense or antisense) was determined by comparison to ORF-Ts [14]. For differential gene expression, the number of RNA-seq reads were calculated for each sense and antisense transcript [14] and merged in to one file. Differentially expressed genes were identified by DESeq2 [36], using corrected *p-*value < 0.05. Convergent overlapping transcripts were excluded from the quantification of antisense transcript levels to avoid potential confusion between sense and antisense transcripts in these cases.

### Availability of supporting data

The data sets supporting the results of this article are available in the GEO repository, http://www.ncbi.nlm.nih.gov/geo/query/acc.cgi?acc=GSE54105.

## Additional files

Additional file 1: Figure S1. 3' end enrichment of Htz1 is not an artefact of antibody cross-reactivity. The α660 antibodies used in this study are highly specific for Htz1 in ChIP [1] but had not been tested in ChIP-seq experiments. To rule out any possibility that the enrichment of Htz1 in the CDS and 3' ends of genes was artefactual, we performed ChIP-seq using this antibody with ChIP extracts generated from an *htz1*Δ strain. The pattern of enrichment in the *htz1Δ* strain was completely different from the wild-type (WT) Htz1 pattern (Pearson correlation, *r* = -0.03) and lacked the peaks seen in the WT sample. **A.** Normalized ChIP signal from *htz1*Δ (upper) and *WT* (lower) cells along chromosome 1, with the coordinates displayed at the bottom. Only the coding regions of a few very highly expressed genes had detectable signal in the *htz1*Δ sample and enrichment at these “hyper-ChIPable” loci is a known artefact in ChIP that is not antibody-related [2]. This confirms that the anti- Htz1 antibody is not precipitating DNA in an Htz1-independent manner. The labeled peaks correspond to: 1, *YAL038W/CDC19*; 2, *tP(UGG)A*; 3, *YAL003W/EFP1*; 4, *tA(UCG)A*; 5, *tL(CAA)A*; 6, *tS(AGA)A*. **B.** Zoomed in views of peaks 1 (*YAL038W/CDC19*; left), and peaks 5 and 6 (*tL(CAA)A, tS(AGA)A;* right) on chromosome 1. ORF and “SGD Other” annotations on the + and – strands are indicated at the right, and key features are labeled for orientation purposes **C.** Htz1 enrichment at three of the genes shown in Figure 3A in the WT and *htz1Δ* strains. There is no enrichment detectable in the *htz1Δ* sample, either at the 5' or 3' ends of the genes. **D.** The average signal at the 3' ends of genes is zero in the *htz1Δ* sample and is significantly lower than the WT sample (*p* = 0; *t*-test). Additional file 2: Figure S2. Coding region Htz1 peaks co-localise with AS transcripts. **A.** Examples of Htz1 peaks in the mid-coding region overlapping with the start of AS transcripts, as indicated by the arrows. Colour coding is as for Figure 3A. **B.** The fraction of Htz1-enriched regions in CDSs associated with AS transcripts is 10% (307 out of 3044). We speculate that other Htz1 peaks in the CDS are also transcript-associated but that these transcripts are not detectable either because they are derived from the sense strand or because they are unstable even in the *rrp6Δ* strain. **C.** Comparison of the number of CDS Htz1 peaks associated with AS transcripts (green line) to the distribution of random CDS regions (black bars) that co-localise with AS transcripts. 307/3044 CDS windows with Htz1 enrichment are associated with AS transcripts. 3044 random windows were drawn from a total set of 38599 windows and randomisation was repeated 100 times to generate the histogram. The association of CDS Htz1 peaks with AS transcripts, although lower than the association of 3' Htz1 peaks with AS transcripts (Figure 3D), is highly significant (*p* = 1.6 x 10-87; Fisher’s exact test). Additional file 3: Figure S3. Effect of Htz1 on sense transcript levels. **A.** Comparison of differential sense transcript levels in *rrp6Δhtz1Δ* versus *rrp6Δ* to Htz1 levels at the 5' ends of genes. Each gene is shown as an open circle, with its 5' Htz1 level measured by ChIP-seq being the y-value and its fold change of expression in the *rrp6Δhtz1Δ* strain shown as its x-value. Significantly up- and down-regulated transcripts are coloured in light blue and pink respectively. **B.** Boxplots of the distributions of 5' Htz1 levels for down- (*n* = 267) and up- (*n* = 255) regulated sense transcripts, show that down-regulated S transcripts are significantly enriched for Htz1 (*****p* ≤ 0.0001 (3.6 x 10-5); two-tailed *t-*test) compared to transcripts whose expression doesn’t change (*n* = 2921). **C.** Actual (solid bars) and expected (hatched bars) numbers of up-/down regulated antisense transcripts with and without 5' Htz1. Down-regulated sense transcripts with 5' Htz1 are significantly more numerous than expected (****p* ≤ 0.001 (4.2 x 10-4); Fisher’s exact test) while up-regulated transcripts are less numerous than expected (*****p* ≤ 0.0001 (2.4 x 10^-14^); Fisher’s exact test). **D.** There is no obvious correlation between enrichment of Htz1 at the 5' end of genes and level of the associated sense transcript. Genes were classified into bins of seven quantiles according to 5' Htz1 level and the distribution of sense transcript levels are plotted for each bin. Additional file 4: Figure S4. Tandem-close genes have higher Htz1 at 3' ends than genes of other arrangements. Distributions of levels of Htz1 at 3' end for tandem-close, tandem-far, convergent-close and convergent-far genes. The amount of Htz1 within a 150bp window upstream of TESs is displayed. Two-tailed *t*-tests show that the level of 3' Htz1 at tandem-close genes is significantly higher relative to levels in the other categories of genes (**** *p* ≤ 0.0001; tandem far *p* = 2.5 x 10^-43^ ; convergent close *p* = 1.5 x 10^-137^; convergent far *p* = 1.8 x 10^-18^;). Additional file 5:
**Table S1.** Correlations between samples. **Table S2.** *S. cerevisiae* genotypes. All strains are derived from Y7092 [3] **Table S3.** Summary of all samples generated in this study and the number of uniquely mapped reads per sample.
