# Supplementary material for: H2A.Z marks antisense promoters and has positive effects on antisense transcript levels in budding yeast
Source: BMC Genomics. 2015 Feb 19;16(1):99. doi: 10.1186/s12864-015-1247-4 (PMC4337092; doi:10.1186/s12864-015-1247-4)
Supplement: Additional file 1: Figure S1. — 3' end enrichment of Htz1 is not an artefact of antibody cross-reactivity. The α660 antibodies used in this study are highly specific for Htz1 in ChIP [1] but had not been tested in ChIP-seq experiments. To rule out any possibility that the enrichment of Htz1 in the CDS and 3' ends of genes was artefactual, we performed ChIP-seq using this antibody with ChIP extracts generated from an htz1Δ strain. The pattern of enrichment in the htz1Δ strain was completely different from the wild-type (WT) Htz1 pattern (Pearson correlation, r = -0.03) and lacked the peaks seen in the WT sample. A. Normalized ChIP signal from htz1Δ (upper) and WT (lower) cells along chromosome 1, with the coordinates displayed at the bottom. Only the coding regions of a few very highly expressed genes had detectable signal in the htz1Δ sample and enrichment at these “hyper-ChIPable” loci is a known artefact in ChIP that is not antibody-related [2]. This confirms that the anti- Htz1 antibody is not precipitating DNA in an Htz1-independent manner. The labeled peaks correspond to: 1, YAL038W/CDC19; 2, tP(UGG)A; 3, YAL003W/EFP1; 4, tA(UCG)A; 5, tL(CAA)A; 6, tS(AGA)A. B. Zoomed in views of peaks 1 (YAL038W/CDC19; left), and peaks 5 and 6 (tL(CAA)A, tS(AGA)A; right) on chromosome 1. ORF and “SGD Other” annotations on the + and – strands are indicated at the right, and key features are labeled for orientation purposes C. Htz1 enrichment at three of the genes shown in Figure 3A in the WT and htz1Δ strains. There is no enrichment detectable in the htz1Δ sample, either at the 5' or 3' ends of the genes. D. The average signal at the 3' ends of genes is zero in the htz1Δ sample and is significantly lower than the WT sample (p = 0; t-test). [file 12864_2015_1247_MOESM1_ESM.pdf]

<u>Gu *et al* Supplemental Figure Legends</u>

Figure S1. 3’ end enrichment of Htz1 is not an artefact of antibody cross-reactivity.

The α660 antibodies used in this study have been previously shown to be highly specific for Htz1 in ChIP [1] but had not been tested across the whole genome in ChIP-seq experiments. To rule out any possibility that the enrichment of Htz1 in the CDS and 3’ ends of genes was artefactual, we performed ChIP-seq using this antibody to immunoprecipitate ChIP extracts generated from an *htz1Δ* strain. The pattern of enrichment in the *htz1Δ* strain was completely different from the wild-type (WT) Htz1 pattern (Pearson correlation, r = -0.03) and lacked the peaks seen in the WT sample.

A. Normalized ChIP signal from *htz1Δ* (upper) and *WT* (lower) cells along chromosome 1, with the coordinates displayed at the bottom. Only the coding regions of a few very highly expressed genes had detectable signal in the *htz1Δ* sample and enrichment at these “hyper-ChIPable” loci is a known artefact in ChIP that is not antibody-related [2]. This confirms that the anti-Htz1 antibody is not precipitating DNA in an Htz1-independent manner. The labeled peaks correspond to: 1, *YAL038W/CDC19*; 2, *tP(UGG)A*; 3, *YAL003W/EFP1*; 4, *tA(UCG)A*; 5, *tL(CAA)A*; 6, *tS(AGA)A*.

B. Zoomed in views of peaks 1 (*YAL038W/CDC19*; left), and peaks 5 and 6 (*tL(CAA)A, tS(AGA)A;* right) on chromosome 1. ORF and “SGD Other” annotations on the + and – strands are indicated at the right, and key features are labeled for orientation purposes.

C. Htz1 enrichment at three of the genes shown in Figure 3A in the WT and *htz1Δ* strains. There is no enrichment detectable in the *htz1Δ* sample, either at the 5’ or 3’ ends of the genes.

D. The average signal at the 3’ ends of genes is zero in the *htz1Δ* sample and is significantly lower than the WT sample (p = 0; *t*-test).

Figure S2: Coding region Htz1 peaks co-localise with AS transcripts.

A. Examples of Htz1 peaks in the mid-coding region overlapping with the start of AS transcripts, as indicated by the arrows. Colour coding is as for Figure 3A.

B. The fraction of Htz1-enriched regions in CDSs associated with AS transcripts is 10% (307 out of 3044). We speculate that other Htz1 peaks in the CDS are also transcript-associated but that these transcripts are not detectable either because they are derived from the sense strand or because they are unstable even in the *rrp6Δ* strain.

C. Comparison of the number of CDS Htz1 peaks associated with AS transcripts (green line) to the distribution of random CDS regions (black bars) that co-localise with AS transcripts. 307/3044 CDS windows with Htz1 enrichment are associated with AS transcripts. 3044 random windows were drawn from a total set of 38599 windows and randomisation was repeated 100 times to generate the histogram. The association of CDS Htz1 peaks with AS transcripts, although lower than the association of 3’ Htz1 peaks with AS transcripts (Figure 3D), is highly significant ($p = 1.6 \times 10^{-87}$; Fisher's exact test).

Figure S3: Effect of Htz1 on sense transcript levels.

A. Comparison of differential sense transcript levels in *rrp6Δhtz1*Δ versus *rrp6Δ* to Htz1 levels at the 5' ends of genes. Each gene is shown as an open circle, with its 5' Htz1 level measured by ChIP-seq being the y-value and its fold change of expression in the *rrp6Δhtz1*Δ strain shown as its x-value. Significantly up- and down-regulated transcripts are coloured in light blue and pink respectively.

B. Boxplots of the distributions of 5' Htz1 levels for down- (n = 267) and up- (n = 255) regulated sense transcripts, show that down-regulated S transcripts are significantly enriched for Htz1 (**** $p \leq 0.0001$ (3.6×10^{-5}); two-tailed *t*-test) compared to transcripts whose expression doesn't change (n = 2921).

C. Actual (solid bars) and expected (hatched bars) numbers of up-/down-regulated antisense transcripts with and without 5' Htz1. Down-regulated sense transcripts with 5' Htz1 are significantly more numerous than expected (*** $p \leq 0.001$ (4.2×10^{-4}); Fisher's exact test) while up-regulated transcripts are less numerous than expected (**** $p \leq 0.0001$ (2.4×10^{-14}); Fisher's exact test).

D. There is no obvious correlation between enrichment of Htz1 at the 5' end of genes and level of the associated sense transcript. Genes were classified into bins of seven quantiles according to 5' Htz1 level and the distribution of sense transcript levels are plotted for each bin.

Figure S4: Tandem-close genes have higher Htz1 at 3' ends than genes of other arrangements.

Distributions of levels of Htz1 at 3' ends for tandem-close, tandem-far,

convergent-close and convergent-far genes. The amount of Htz1 within a 150bp window upstream of TESs is displayed. Two-tailed *t*-tests show that the level of 3' Htz1 at tandem-close genes is significantly higher relative to levels in the other categories of genes (**** $p \leq 0.0001$; tandem far $p = 2.5 \times 10^{-43}$; convergent close $p = 1.5 \times 10^{-137}$; convergent far $p = 1.8 \times 10^{-18;}$)

Supplemental Tables.

Table S1. Correlations between samples.

A. Pearson correlations between ChIP-seq experiments.

Genotype	*WT (772) replicate 1*	*WT (772) replicate 2*	*rrp6Δ (884) replicate 1*	*rrp6Δ (884) replicate 2*
WT (772) replicate 1	1	0.99	0.94	0.97
WT (772) replicate 2		1	0.95	0.97
rrp6Δ (884) replicate 1			1	0.96
rrp6Δ (884) replicate 2				1

B. Pearson correlations between biological replicates for RNA-seq experiments.

Genotype	*WT rep1*	*WT rep2*	*htz1Δ rep1*	*htz1Δ rep2*	*rrp6Δ rep1*	*rrp6Δ rep2*	*rrp6Δhtz1Δ rep1*	*rrp6Δhtz1Δ rep2*
WT (772) rep1	1	0.99	0.98	0.98	0.96	0.95	0.93	0.93
WT (772) rep2		1	0.99	0.99	0.96	0.96	0.94	0.94
htz1Δ (773) rep1			1	0.99	0.95	0.94	0.94	0.94
htz1Δ (773) rep2				1	0.96	0.96	0.95	0.96
rrp6Δ (884) rep1					1	0.99	0.96	0.96
rrp6Δ (884) rep2						1	0.96	0.97
rrp6Δhtz1Δ (885) rep1							1	0.99
rrp6Δhtz1Δ (885) rep2								1

Table S2. ***S. cerevisiae* genotypes**. All strains are derived from Y7092 [3].

Name	Genotype	Reference
CMY772	*MATα can1Δ::STE2pr-SP_his5 lyp1Δ his3Δ1 ura3Δ0 met15Δ0 HTZ1-HpH*	This study
CMY773	*MATα can1Δ::STE2pr-SP_his5 lyp1Δ his3Δ1 ura3Δ0 met15Δ0 htz1Δ::HpH*	This study
CMY884	*MATα can1Δ::STE2pr-SP_his5 lyp1Δ his3Δ1 ura3Δ0 met15Δ0 HTZ1-HpH rrp6Δ::KanMX*	This study
CMY885	*MATα can1Δ::STE2pr-SP_his5 lyp1Δ his3Δ1 ura3Δ0 met15Δ0 htz1Δ::HpH rrp6Δ::KanMX*	This study

Table S3. Summary of all samples generated in this study and the number of uniquely mapped reads per sample.

**Experiment**	**Strain**	**# uniquely mapped reads**
ChIP-seq	WT (CMY772) replicate 1	14,697,299
ChIP-seq	WT (CMY772) replicate 2	13,231,718
ChIP-seq	*htz1Δ* (CMY773)	8,661,572
ChIP-seq	*rrp6Δ* (CMY884) replicate 1	11,977,811
ChIP-seq	*rrp6Δ* (CMY884) replicate 2	14,502,522
ChIP-seq	Input DNA	15,300,627
RNA-seq	WT (CMY772) replicate 1	31,405,138
RNA-seq	WT (CMY772) replicate 2	23,038,629
RNA-seq	htz1Δ (CMY773) replicate 1	23,119,171
RNA-seq	*htz1Δ* (CMY773) replicate 2	32,566,862
RNA-seq	*rrp6Δ* (CMY884) replicate 1	57,034,365
RNA-seq	*rrp6Δ* (CMY884) replicate 2	39,591,473
RNA-seq	*rrp6Δhtz1Δ* (CMY885) replicate 1	47,638,662
RNA-seq	*rrp6Δhtz1Δ* (CMY885) replicate 2	27,925,445

<u>Supplemental References.</u>

1. Millar CB, Xu F, Zhang K, Grunstein M: **Acetylation of H2AZ Lys 14 is associated with genome-wide gene activity in yeast**. *Genes Dev* 2006, **20**:711–722.

2. Teytelman L, Thurtle DM, Rine J, van Oudenaarden A: **Highly expressed loci are vulnerable to misleading ChIP localization of multiple unrelated

proteins. *Proceedings of the National Academy of Sciences* 2013, **110**:18602–18607.

3. Tong AHY, Tong AHY, Boone C, Boone C: **High-Throughput Strain Construction and Systematic Synthetic Lethal Screening in**. In *Methods in Microbiology*. *Volume 36*. Elsevier; 2007:369–707. [*Methods in Microbiology*]
