# Supplementary figures and images for: H2A.Z marks antisense promoters and has positive effects on antisense transcript levels in budding yeast
Source: BMC Genomics. 2015 Feb 19;16(1):99. doi: 10.1186/s12864-015-1247-4 (PMC4337092; doi:10.1186/s12864-015-1247-4)

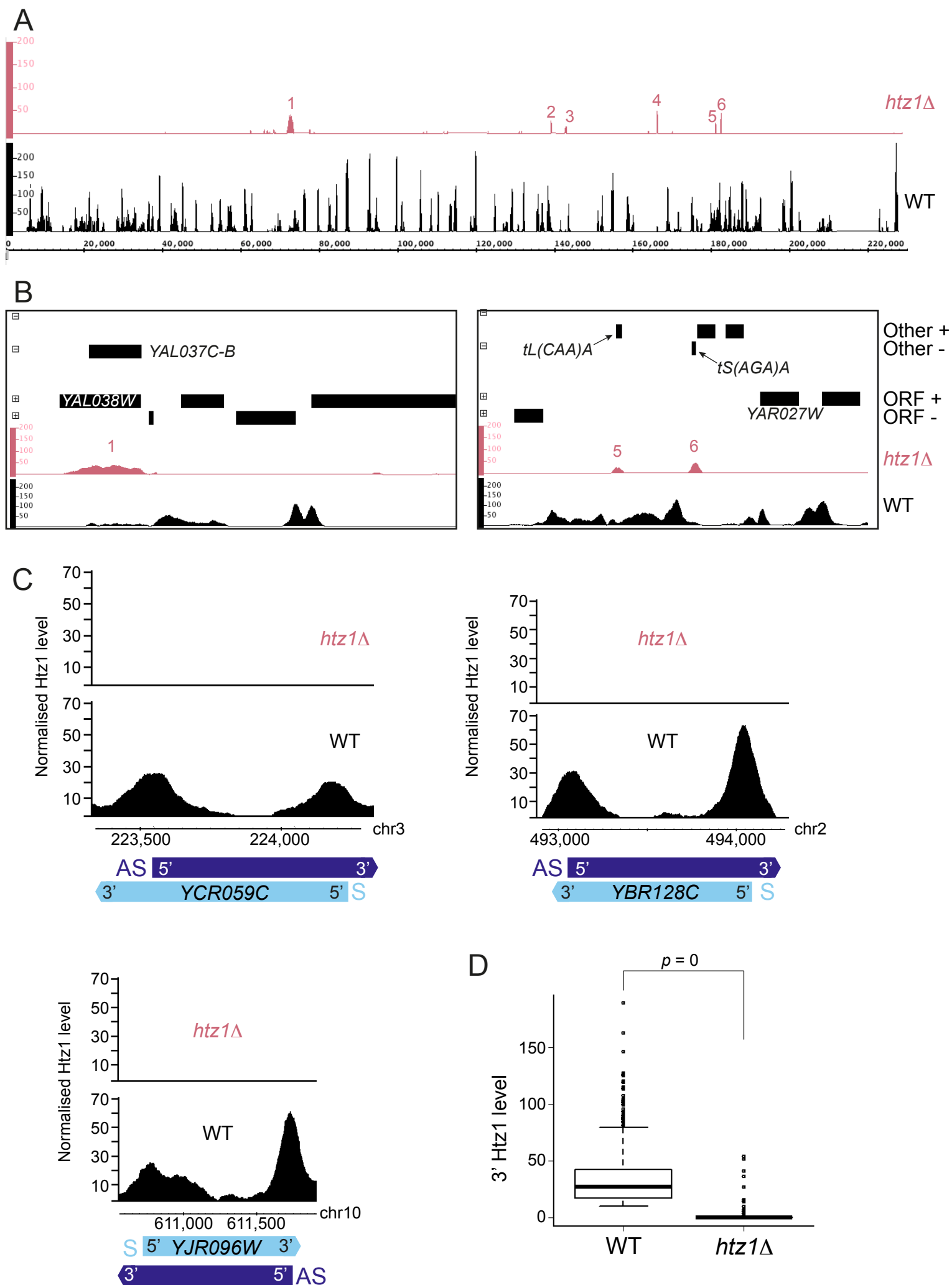

Supplement: Additional file 2: Figure S2. — Coding region Htz1 peaks co-localise with AS transcripts. A. Examples of Htz1 peaks in the mid-coding region overlapping with the start of AS transcripts, as indicated by the arrows. Colour coding is as for Figure 3A. B. The fraction of Htz1-enriched regions in CDSs associated with AS transcripts is 10% (307 out of 3044). We speculate that other Htz1 peaks in the CDS are also transcript-associated but that these transcripts are not detectable either because they are derived from the sense strand or because they are unstable even in the rrp6Δ strain. C. Comparison of the number of CDS Htz1 peaks associated with AS transcripts (green line) to the distribution of random CDS regions (black bars) that co-localise with AS transcripts. 307/3044 CDS windows with Htz1 enrichment are associated with AS transcripts. 3044 random windows were drawn from a total set of 38599 windows and randomisation was repeated 100 times to generate the histogram. The association of CDS Htz1 peaks with AS transcripts, although lower than the association of 3' Htz1 peaks with AS transcripts (Figure 3D), is highly significant (p = 1.6 x 10^-87^; Fisher’s exact test). [file 12864_2015_1247_MOESM2_ESM.pdf]

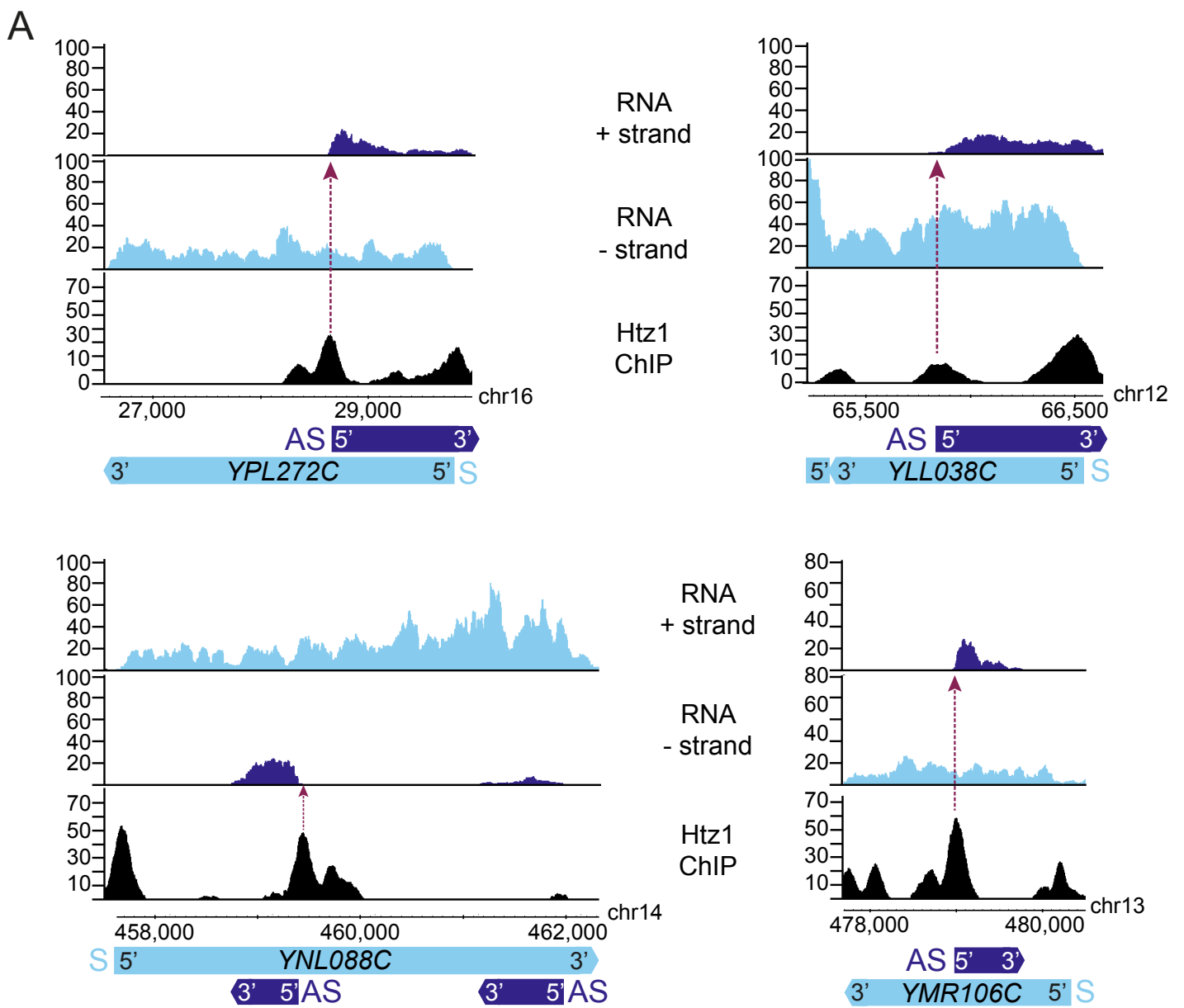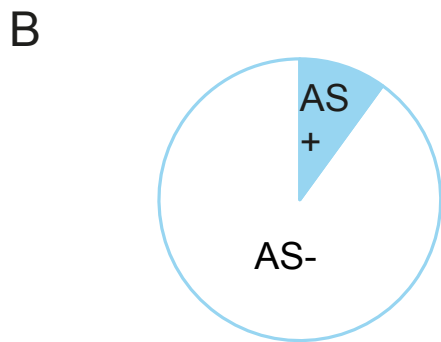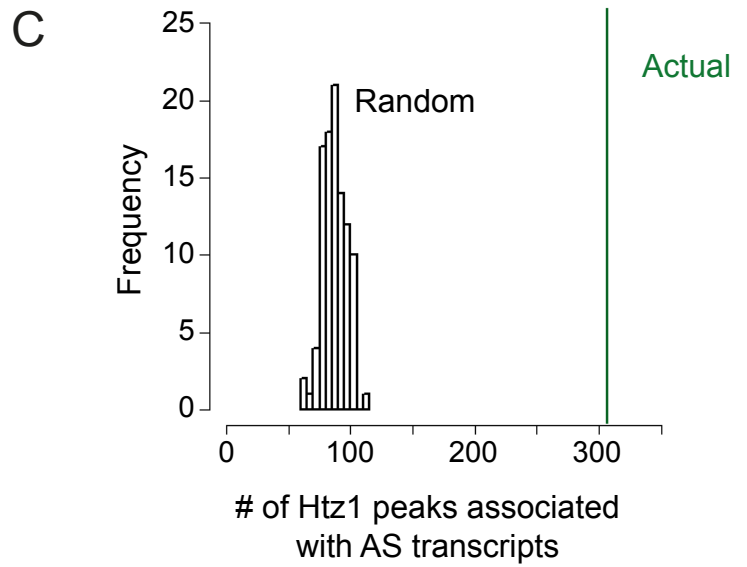

Supplement: Additional file 3: Figure S3. — Effect of Htz1 on sense transcript levels. A. Comparison of differential sense transcript levels in rrp6Δhtz1Δ versus rrp6Δ to Htz1 levels at the 5' ends of genes. Each gene is shown as an open circle, with its 5' Htz1 level measured by ChIP-seq being the y-value and its fold change of expression in the rrp6Δhtz1Δ strain shown as its x-value. Significantly up- and down-regulated transcripts are coloured in light blue and pink respectively. B. Boxplots of the distributions of 5' Htz1 levels for down- (n = 267) and up- (n = 255) regulated sense transcripts, show that down-regulated S transcripts are significantly enriched for Htz1 (****p ≤ 0.0001 (3.6 x 10^-5^); two-tailed t-test) compared to transcripts whose expression doesn’t change (n = 2921). C. Actual (solid bars) and expected (hatched bars) numbers of up-/down regulated antisense transcripts with and without 5' Htz1. Down-regulated sense transcripts with 5' Htz1 are significantly more numerous than expected (***p ≤ 0.001 (4.2 x 10^-4^); Fisher’s exact test) while up-regulated transcripts are less numerous than expected (****p ≤ 0.0001 (2.4 x 10^-14^); Fisher’s exact test). D. There is no obvious correlation between enrichment of Htz1 at the 5' end of genes and level of the associated sense transcript. Genes were classified into bins of seven quantiles according to 5' Htz1 level and the distribution of sense transcript levels are plotted for each bin. [file 12864_2015_1247_MOESM3_ESM.pdf]

A

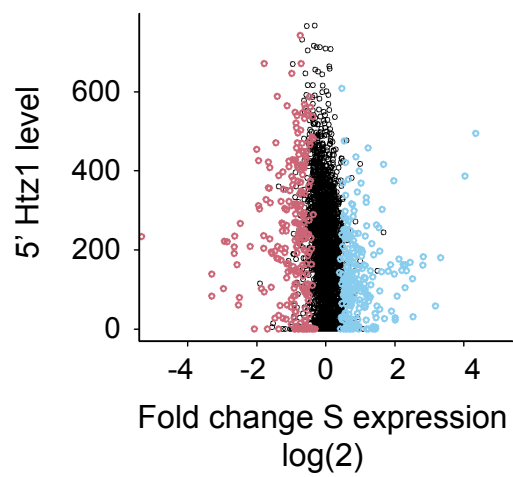

B

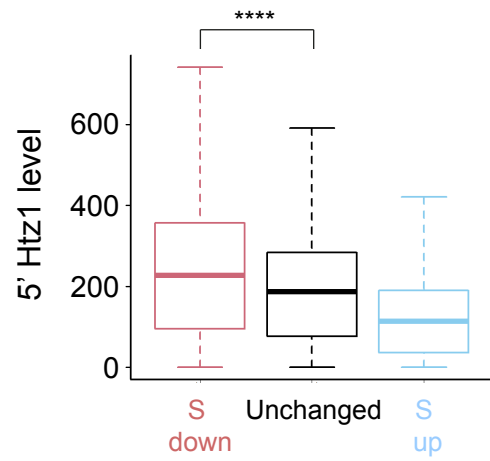

C

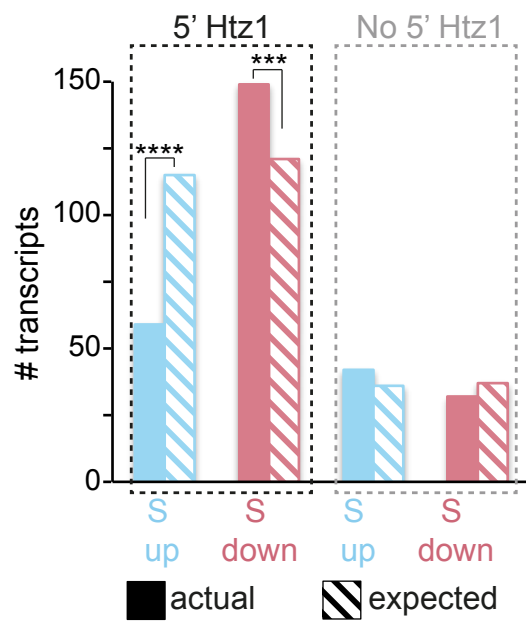

D

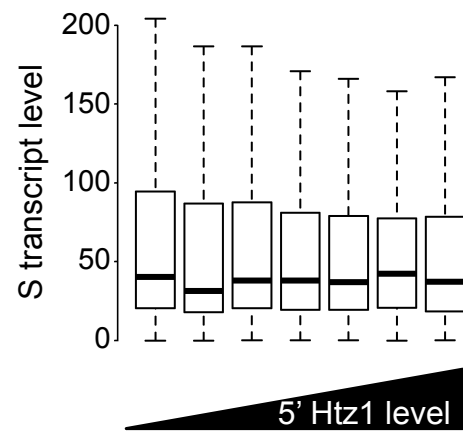

Gu et al Figure S3

Supplement: Additional file 4: Figure S4. — Tandem-close genes have higher Htz1 at 3' ends than genes of other arrangements. Distributions of levels of Htz1 at 3' end for tandem-close, tandem-far, convergent-close and convergent-far genes. The amount of Htz1 within a 150bp window upstream of TESs is displayed. Two-tailed t-tests show that the level of 3' Htz1 at tandem-close genes is significantly higher relative to levels in the other categories of genes (**** p ≤ 0.0001; tandem far p = 2.5 x 10^-43^ ; convergent close p = 1.5 x 10-137; convergent far p = 1.8 x 10^-18^;). [file 12864_2015_1247_MOESM4_ESM.pdf]

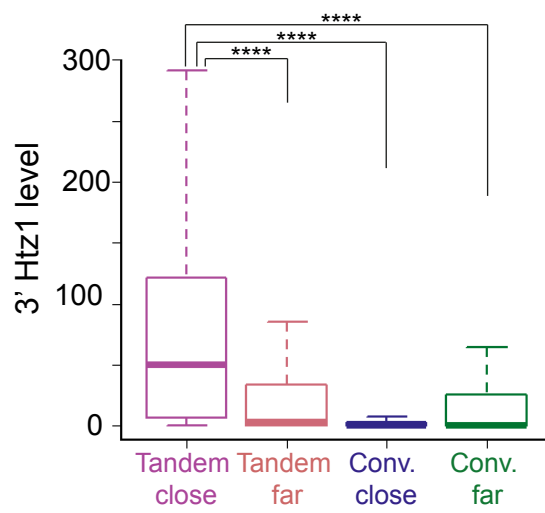

Gu et al Figure S4

Supplement: Additional file 5: — Table S1. Correlations between samples. Table S2. S. cerevisiae genotypes. All strains are derived from Y7092 [3] Table S3. Summary of all samples generated in this study and the number of uniquely mapped reads per sample. [file 12864_2015_1247_MOESM5_ESM.pdf]
